# Life times of metastable states guide regulatory signaling in transcriptional riboswitches

**DOI:** 10.1038/s41467-018-03375-w

**Published:** 2018-03-05

**Authors:** Christina Helmling, Dean-Paulos Klötzner, Florian Sochor, Rachel Anne Mooney, Anna Wacker, Robert Landick, Boris Fürtig, Alexander Heckel, Harald Schwalbe

**Affiliations:** 1 Institute for Organic Chemistry and Chemical Biology Center for Biomolecular Magnetic Resonance (BMRZ), Johann Wolfgang Goethe-Universität, Max-von-Laue-Straße 9, 60438 Frankfurt, Germany; 2 Institute for Organic Chemistry and Chemical Biology, Johann Wolfgang Goethe-University Frankfurt, Max-von-Laue-Straße 9, 60438 Frankfurt, Germany; 3 Department of Biochemistry, University of Wisconsin–Madison, Madison, WI 53706 USA

## Abstract

Transcriptional riboswitches modulate downstream gene expression by a tight coupling of ligand-dependent RNA folding kinetics with the rate of transcription. RNA folding pathways leading to functional ON and OFF regulation involve the formation of metastable states within well-defined sequence intervals during transcription. The kinetic requirements for the formation and preservation of these metastable states in the context of transcription remain unresolved. Here, we reversibly trap the previously defined regulatory relevant metastable intermediate of the *Mesoplasma florum* 2′-deoxyguanosine (2′dG)-sensing riboswitch using a photocaging-ligation approach, and monitor folding to its native state by real-time NMR in both presence and absence of ligand. We further determine transcription rates for two different bacterial RNA polymerases. Our results reveal that the riboswitch functions only at transcription rates typical for bacterial polymerases (10–50 nt s^−1^) and that gene expression is modulated by 40–50% only, while subtle differences in folding rates guide population ratios within the structural ensemble to a specific regulatory outcome.

## Abstract (web summary)

Riboswitches are RNA-based regulatory elements, which regulate downstream gene expression by binding of small molecular weight ligands. Here the authors demonstrate the molecular mechanism of a transcriptional riboswitch that integrates changes in transcription rates, metabolite concentration, and kinetic on- and off-rates of ligand binding.

## Introduction

Riboswitches selectively bind metabolites to control the transcription or translation of metabolite-associated genes. In general, riboswitch function is described as a ligand-dependent switch between two mutually exclusive conformations to either permit or repress gene expression^1,2^. For riboswitches that regulate gene expression on the level of translation, reversible conformational switching allows RNAs to adopt distinct ligand-dependent functional folds at equilibrium during multiple rounds of translation. As a consequence, RNA synthesis and the cellular response to changes in metabolite concentration are separated in time. The functional relevant switch underlying translational riboswitches modulate ribosome access to the SD sequence^3–6^, but also more complex regulation mechanisms have been discovered^7^.

In contrast, control of gene expression by premature termination of transcription inevitably requires temporal coupling of RNA synthesis and conformational switching. Riboswitches utilize rho-independent transcriptional termination^8,9^. Under these conditions, the nascent transcript can either adopt a sequence-conserved terminator conformation that disrupts the polymerase complex or form one or several alternative, so-called antiterminator conformations that avoid termination. The metabolite concentration EC_50_, at which riboswitches operate, often exceeds the K_D_ values for ligand binding by at least one order of magnitude^3,10–14^. This observation indicates that fast folding of functional states during well-defined transcription intervals is important for riboswitch function.

**Fig. 1:**
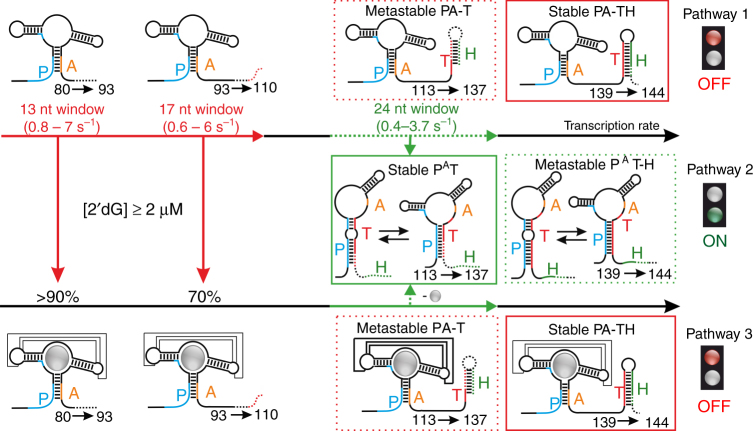
Non-equilibrium model for the co-transcriptional conformational switch of the 2′dG-sensing riboswitch. Regulatory function by transcriptional riboswitches is based on a ligand-dependent interplay between the four sequence segments P (5′-aptamer strand), A (aptamer-stabilizing strand), T (switching strand), and H (terminator strand)^15^. Previously derived transcription intervals for folding events involving a stabilization of the aptamer domain via the P–A interaction or switch in strand interaction (A–T interaction) are highlighted in red (ligand binding) and green (ON state folding), respectively. Directly after transcription of the aptamer domain, 2′dG can bind during the synthesis of nts 80–110 (30 nt window), with a continuous decrease in the ligand binding efficiency from >90% to 70% between nucleotides 93 and 110. Continuation of transcription beyond nt 113 transforms both the ligand-bound and ligand-free aptamer domains (PA–T) into metastable states (dashed red box). Folding to the regulatory ON state (P^A^T, solid green box) can only occur during synthesis of nucleotides 113–137. At the regulatory decision point, folded P^A^T–H states are metastable (dashed green box), while the PA–TH conformation (solid red box) represents the lowest free energy state in both absence and presence of ligand. Metastable states are highlighted by dashed boxes and lowest free energy states by solid boxes

In a previous study, we investigated the landscape of the thermodynamically stable states adopted in either the presence or absence of metabolite for mRNA transcripts of increasing chain length^15^. The ligand-dependent conformational rearrangement underlying riboswitch function typically involves four distinct sequence segments P, A, T, and H as depicted in Fig. 1. In the 2′dG riboswitch, an mRNA containing 80 nucleotides is the first transcript that can form the interaction between strands P and A. Ligand binding can occur directly after the aptamer domain is transcribed. Interestingly, ligand can also bind to the growing mRNA up to nucleotides 80–113 nt, but the stability of these mRNA-ligand complexes and thus the population of ligand-bound states continuously decrease during the addition of nts 90–113 from >90% to 70%. As strand T is transcribed, the PA interaction becomes metastable (PA–T) in both the ligand-bound and ligand-free form. In the equilibrium model, folding from PA–T to the antiterminator conformation P^A^T concomitantly occurs during transcription of nucleotides 113 to 137. Previous studies assume ligand binding within the first transcription interval (nts 80–110) to stabilize the PA interaction sufficiently to inhibit an interaction with strand T and to ensure OFF function, but it remains uncertain how the P^A^T state is selected from only one metastable state in the context of transcription. P^A^T states adopted within the second transcription interval (in green) become metastable as strand H is transcribed (P^A^T–H) during synthesis of nts 139 to 144 in both, absence or presence of ligand. Within this short period during transcription, the terminator conformation PA–TH represents the most stable state. It follows that deciphering the change of kinetics of antiterminator formation (P^A^T) depending on the absence and presence of ligand represents the most important information regarding regulation.

According to the equilibrium model, the effects of ligand binding on the overall fold adopted at a specific transcript length is insignificant. Rather, formation of metastable intermediate states as important navigators of the regulatory outcome suggests a correlation between the life times of these states and transcription rates to be critical for riboswitch function. Here, we unravel these kinetic requirements to achieve and to maintain a ligand-dependent reversal of population ratios of functional OFF and ON states during transcription. On the example of the central metastable PA–T conformation of the 2′dG riboswitch, we demonstrate that ligand binding does not induce major changes in equilibration kinetics of the PA–T state, but slows refolding to allow the polymerase to bypass the metastable transcription barrier for the majority of nascent transcripts.

## Results

### Reversible trapping of the metastable PA–T conformation

To monitor antiterminator (P^A^T) folding, we trapped the PA conformation of the 121 mer transcriptional intermediate by incorporating photolabile protection groups in strand T. These protection groups prevent antiterminator helix PT formation and allow monitoring of folding from PA to PT after photolysis by light-induced real-time NMR as reported previously^16–18^. The 121 nt transcriptional intermediate forms a complete antiterminator helix PT at equilibrium. While refolding from PA–T to P^A^T is initiated at nucleotide 113, segment 113–121 only contains four weak base pairs within helix PT, and, therefore, refolding kinetics from PA–T to P^A^T are most accurately described at length 121 on average.

**Fig. 2:**
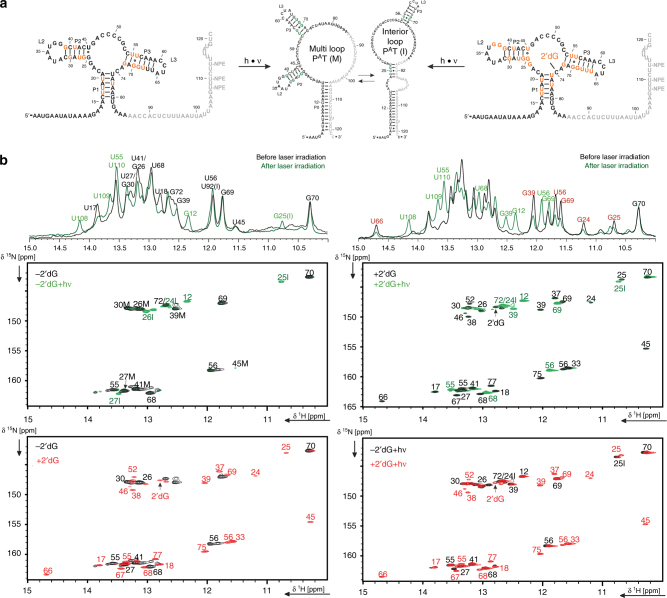
Structural integrity of caged dGsw^121^ before and after laser irradiation. **a** Secondary structures of dGsw^121^ in presence and absence of ligand, before photolysis (PA–T) and after photolysis to form the antiterminator conformations P^A^T(M) and P^A^T(I). Aptamer domain imino protons detectable by NMR are highlighted in orange. Antiterminator imino protons detectable by NMR are highlighted in green. **b** Overlay of 1D NMR spectra of caged dGsw^121^ before (black) and after (green) laser irradiation in absence (left) and presence (right) of 2 eq. of ligand  and combinations of overlay of ^1^H,^15^N -TROSY of dGsw^121^ in absence (−2′dG) or presence of 2 eq. ligand ( + 2′dG), and before and after ( + hν) laser irradiation. The spectra were recorded on 100 µM NMR samples with 6 eq. of Mg^2+^ at 800 MHz and 298 K. Characteristic imino proton signals to monitor the formation of P^A^T are highlighted in green. Characteristic imino proton signals to monitor aptamer dissociation in the presence of ligand are highlighted in red

Photocaged dGsw^121^ (dGsw^121,caged^) was prepared by enzymatic splinted ligation and contained three NPE protection groups at the C4 atoms in the nucleobases of Uracil residues 108, 110, and 112 (Fig. 2a, Supplementary Fig. 1). In addition to steric hindrance, these NPE protection groups introduce a tautomeric shift at the Watson–Crick base pairing site to prevent base pairing. The RNA fragment comprising nucleotides 1–85 was transcribed in ^15^N-G/U isotope labeled form. The NPE-protected smaller fragment comprising nucleotides 86–121 was chemically synthesized (Supplementary Fig. 2, Table 1). We analyzed dGsw^121,caged^ by NMR-spectroscopy to verify that trapping the PA fold in the caged construct was successful. Figure 2b shows an overlay of NMR spectra before and after photolysis, while in the ^15^N-correlation spectra only the ^15^N-labeled fragment (nts 0–85) is detectable. In dGsw^121,caged^, the conformation of the aptamer domain (PA–T) is maintained as evidenced by characteristic imino proton reporter signals, in particular U17 and U18^19^. Irradiation at 355 nm releases native dGsw^121^ and subsequently induces formation not of a single, but of two interconverting antiterminator conformations (P^A^T(I)) and (P^A^T(M)), identified by helix PT reporter signals G12, U108, U109, and G25I. In the presence of ligand, the antiterminator conformations P^A^T folds only to ~80%, in agreement with the static experiments^15^. In the ligand-free form, chemical shifts of helices P2 and P3 are identical in either the aptamer PA or antiterminator P^A^T conformations, while those signals can be distinguished in the ligand-bound form due tertiary interactions induced by ligand binding.

### Life times of the metastable PA–T state by real-time NMR

**Fig. 3:**
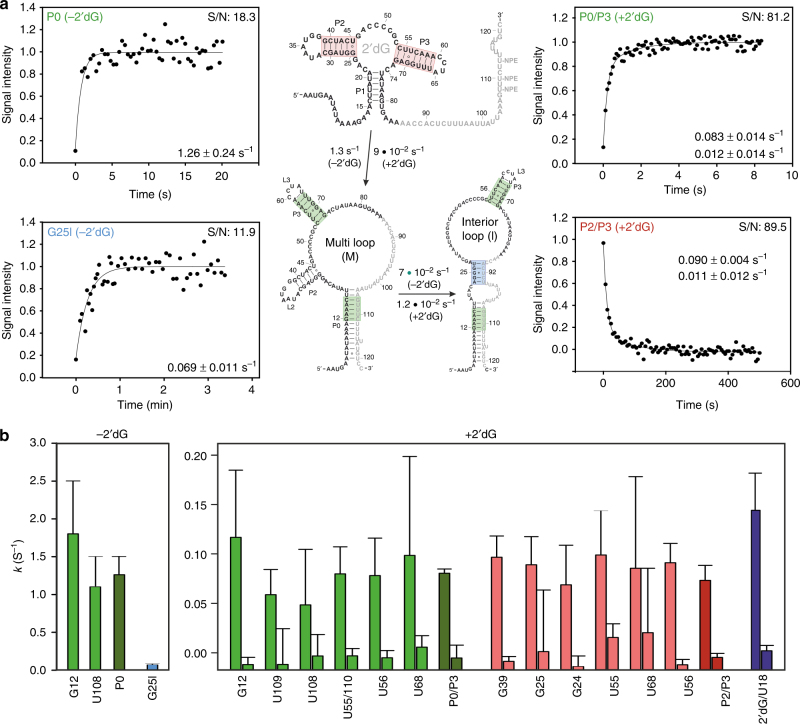
Kinetic traces for ON state folding. **a** Averaged kinetic traces for antiterminator P^A^T(M) formation in presence and absence of ligand (green), kinetic trace for antiterminator P^A^T(I) formation derived from the imino proton reporter signal G25I (blue), and averaged kinetic trace for aptamer dissociation in the presence of ligand (red). Dissociation and association of helical segments monitored by real-time NMR are color coded accordingly in the secondary structure depiction on the right. **b** Individual rates derived from transients shown in (**a**) and Supplementary Fig. 3. Green bars indicate rates of P0 and P3 formation in P^A^T(I) and P^A^T(M) conformations, with the dark green bar corresponding to averaged rates for complete helix formation. The blue bar shows kinetic rates for P^A^T(I) formation only. Red bars correspond to helix P2 and P3 dissociation in the ligand-bound state and dark red to the averaged dissociation of helix P2 and P3. The purple bar corresponds to cooperative dissociation of both the ligand 2′dG and U18 (P1). Errors correspond to the s.d. of the fit. S/N values shown correspond to the S/N ratio of the latest data point

We obtained kinetic traces by real-time NMR at the physiological temperature of the bacterium *M*. *florum* (25 °C). Rates for individual base pairs were determined (Supplementary Fig. 3), utilizing F-statistics to check for statistical significance (Supplementary Table 2). Rapid formation of P^A^T in the absence of ligand required averaging of transients obtained from individual imino protons to yield a final rate for helix formation with improved accuracy (Fig. 3a). We can confirm the validity of this approach by the larger number of kinetic traces with improved S/N obtained for the ligand-bound form (Fig. 3b), where the average rate for helix P2/P3 formation accurately reflects rates of individual bases. In the absence of ligand, P^A^T(M) folds at a rate of ~1.3 s^−1^. The second conformation P^A^T(I) could be monitored by the characteristic and isolated signal G25I, and folds at 10-fold slower rates of 7*10^−2^ s^−1^. In the ligand-bound form, the difference in chemical shift of helices P2/P3 in PA–T and P^A^T allows monitoring of aptamer dissociation in addition to PT formation. The ligand-bound PA conformation folds to P^A^T(M) at rates of ~9*10^−2^ s^−1^ with a second slower rate in the order of ~1*10^−2^ s^−1^, which we assign to re-equilibration to P^A^T(M) after formation of P^A^T(I).

The analysis of the kinetics reveals three relevant functional pathways as depicted in Fig. 1. Folding of P^A^T(M) in the absence of ligand (pathway 2) is on the same timescale (1.3 s^−1^) as bacterial polymerases require to synthesize the subsequent 24 nucleotides (0.4–3.7 s^−1^)^20–23^. The synchronization of transcription with folding rates that are unimolecular and thus concentration independent and sufficiently long life-time of the metastable state ensures ON state function. In contrast, ligand binding slows down folding from the PA conformation to the P^A^T(M) conformation to a rate of ~0.1 s^−1^, and, thus, below transcription rates (pathway 3). Hence, in the presence of ligand, antiterminator formation for transcript length for which it represents the stable fold (113–137 nts) is too slow, and, thus, OFF state function is triggered. Further, equilibration between P^A^T(M) and P^A^T(I), occurs on the same timescale as antiterminator formation in the presence of ligand, and, therefore, cannot compete with transcription rates. Therefore, our kinetic experiments reveal that only one of the two antiterminator conformations (P^A^T(M)) represents the regulatory decisive ON state, and, thus, demonstrate a kinetic selection of only a single functionally relevant conformation in the structural ensemble.

### Ligand binding kinetics

**Fig. 4:**
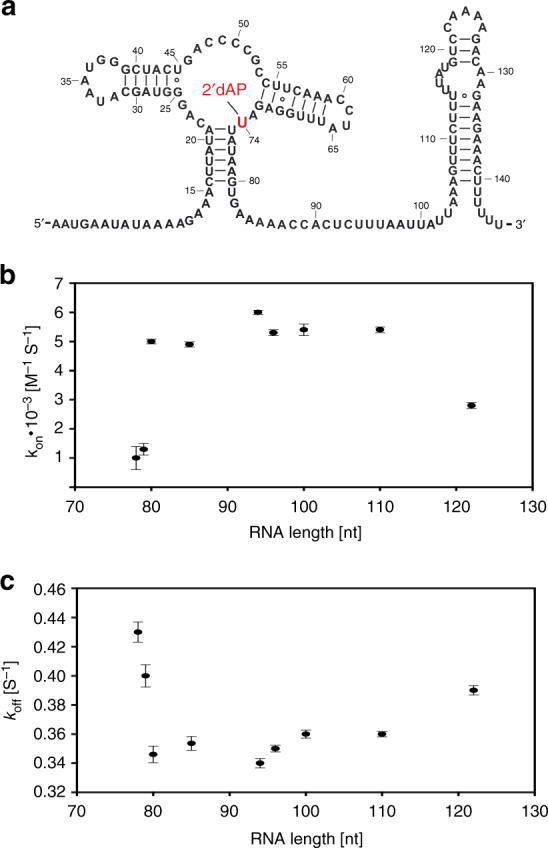
Ligand binding kinetics. **a** Secondary structure of dGsw^C74U^ highlighting the C to U mutation at position 74 in red to accommodate the fluorescence ligand analog 2-aminopurine-2′deoxyriboside (2′dAP). **b** Final k_on_ and **c** k_off_ rates of 2′dAP binding to dGsw^C74U^ derived from data shown in Supplementary Fig. 4 and Table 3 at varying transcript lengths. Errors correspond to the s.d. of the fit

OFF state function depends on ligand binding kinetics and subsequent RNA folding. We further determined the kinetics for transcripts of different chain length by stopped-flow spectroscopy with a surrogate fluorescence ligand 2-aminopurine 2′-deoxyriboside (2′dAP) to investigate whether the ligand association rates (k_on_) remain constant within the relevant transcription interval. To facilitate binding of 2′dAP, the ligand selectivity was shifted from G to A by mutating the Watson–Crick ligand recognition site C74 to U74 (dGsw^C74U^), a common approach applied for purine riboswitches (Fig. 4a)^24,25^. Homogeneous binding of 2′dAP to dGsw^C74U^ was verified by NMR and k_on_ rates were obtained for 9 different transcriptional intermediates (Supplementary Fig. 4 and Table 3). The results show that ligand binding kinetics are virtually identical for constructs that adopt a stable PA fold based on static measurements (Fig. 4b, transcript lengths 80–113 nt). Truncated P1 constructs as well as antiterminator constructs exhibit a reduced k_on_ rate by a factor of 5 and 2, respectively. Since K_D_ values of 2′dAP binding to dGsw^C74U^ (60 µM) exceeds the K_D_ of 2′dG binding to dGsw (250 nM), the determined k_off_ for 2′dAP of 0.35 s^−1^ provides an upper limit for k_off_ of 2′dG.

Analysis of ligand binding by stopped-flow spectroscopy shows that ligand binding kinetics remains constant for stably formed aptamer domains regardless of transcript length. Due to decline in the ligand-bound equilibrium population between transcript length 90 and 110, the amount of structures accumulating within pathway 3 depends on cellular ligand concentration and corresponding kinetics of ligand association. Therefore, at saturating ligand concentration, the bound state (pathway 3) will be populated in a range of 70–100% by the time the polymerase has reached nucleotide 113. Equilibration can take place during transcription of nts 90–110, transcribing at rates of 0.5-4.5 s^−1^. From determined k_off_ rates, we conclude that once bound, the ligand will not dissociate during the transcription interval for ligand binding.

### Simulations of co-transcriptional folding

**Fig. 5:**
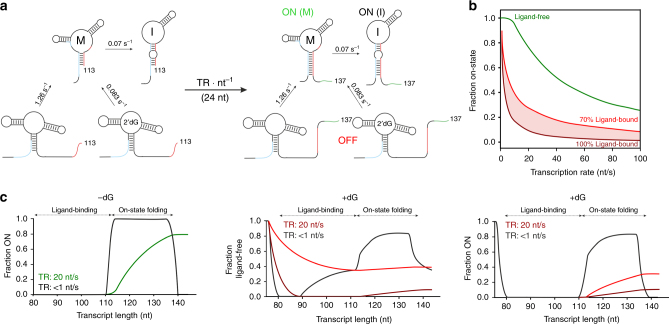
Simulations of co-transcriptional folding. **a** Schematic representation of interconverting transcriptional intermediates between transcript lengths 113 and 137 nt and corresponding refolding rates derived from real-time NMR experiments. **b** Fraction of ON state obtained at nucleotide 137 depending on the transcription rate. Individual simulations were performed for interconverting transcriptional intermediates between transcript lengths 113 and 137 nt based on interconversion rates derived from real-time NMR experiments. The following three instances were simulated: the ligand is not bound to the aptamer domain before nucleotide 113 is synthesized (ligand-free, green); ligand binding is completed by 70% (70% ligand-bound, light red) or 100% (100% ligand-bound, dark red). **c** Comparison of co-transcriptional folding at a transcription rate of 20 nt s^−1^ to equilibrium structures assuming the polymerase stalls at each nucleotide (<1 nt s^−1^). Equilibrium structures of transcriptional intermediates were determined previously (black trace)^15^. For a transcription rate of 20  nt s^−1^, simulations of co-transcriptional folding between transcript lengths 113 and 137 nt were fit into the corresponding graphs. The state adopted at nucleotide 137 is assumed to be maintained until the regulatory decision point. The ligand-free fraction between nucleotides 78 and 110 corresponds to a single-exponential decay resulting in either a 70% ligand-bound population at nucleotide 113 (~0.6 s^−1^) or a 100% ligand-bound population at nucleotide 90 (~2.3 s^−1^)

We determined all relevant conformational transition kinetics along and between pathways 1, 2, and 3 by real-time NMR and stopped-flow spectroscopy, allowing us to calculate the kinetics of the switch between different regulatory pathways (Fig. 5). We performed simulations of the conformational transition from PA–T to P^A^T between transcript lengths 113 and 137 at different transcription rates (0–100 nt s^−1^) (Supplementary Fig. 5) and derived the fraction of ON state after this transcription interval (Fig. 5a). Equilibration from the metastable P^A^T-H state to the PA–TH state can be initiated once nucleotide 139 emerges from the transcribing polymerase, one nucleotide prior to the regulatory signal. Therefore, we consider nucleotide 137 to represent the latest possible regulatory decision point. The fraction of ON state obtained from individual simulations at different transcription rates is shown in Fig. 5b for (i) 2′dG is not bound (in green) and (ii) 2′dG is bound by 70–100% (in red) to the PA–T conformation by the time nt 113 is synthesized. Figure 5c visualizes the effective ligand-induced kinetic delay of PT formation at a transcription rate of 20 nt s^−1^ that offsets equilibrium populations previously determined^15^. Kinetic traces between nucleotides 113 and 137 correspond to individual simulations derived from Supplementary Fig. 6. In the presence of ligand, the simulations further distinguish between OFF state (pathways 1 + 3) and ligand-bound state (pathways 3), where the OFF state includes ligand-free folded aptamer domains that also terminate transcription.

The simulations show that the riboswitch functions only at transcription rates of 10–50 nt s^−1^, a value that matches those typical for bacterial polymerases (Fig. 5b). Slow transcription rates <10 nt s^−1^ extend the time interval for ON state folding to a point, where ligand binding can no longer delay ON state folding sufficiently. At transcription rates exceeding 70 nt s^−1^, ON state folding in both absence and presence of ligand cannot compete with the rate of transcription. In this system of competing kinetic requirements, a maximum regulation efficiency of 40–50% is achieved only at transcription rates of 10–50 nt s^−1^. Comparison of static and kinetic experiments shows that the equilibration kinetics of metastable states neither lead to complete formation of the functional ON state from pathway 1 or preservation of the functional OFF state in pathway 3 during transcription.

### In vitro transcription assays

We performed single-round in vitro transcription assays in the absence and presence of ligand using both, *E*. *coli* and *B*. *subtilis* DNA-dependent RNA polymerases (RNAP) to (i) experimentally validate that the riboswitch functions only in a defined window of transcription rates and to (ii) assess whether the co-transcriptional folding pathway of the riboswitch is sensitive to the transcription properties of the polymerases from different organisms. We determined the dependence of transcription rates on temperature and NTP concentrations. We further determined the regulation efficiency by quantifying the amount of 235 nt fragments elongated past the terminator sequence.

**Fig. 6:**
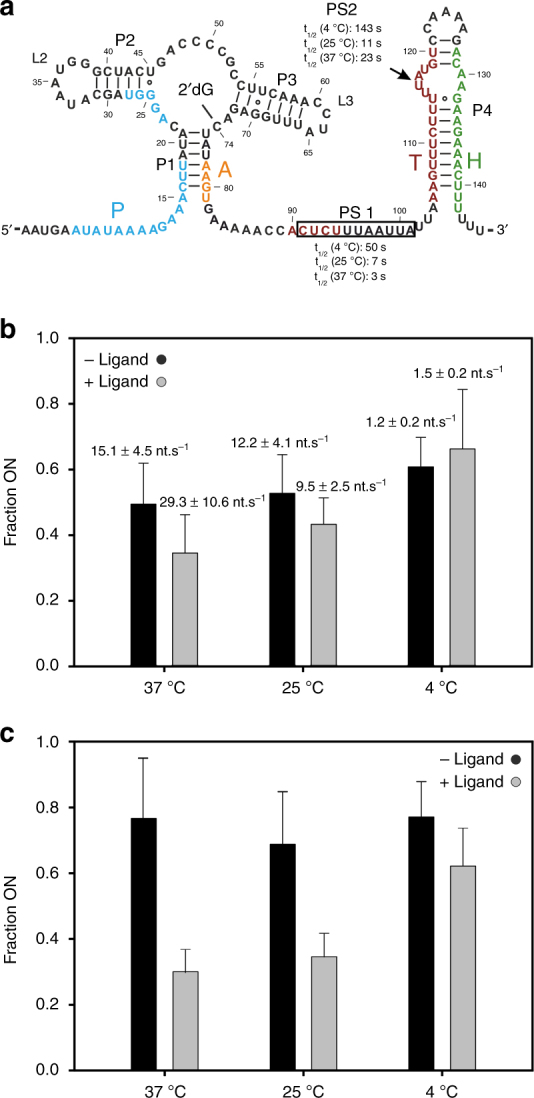
Fractions of ON state derived by in vitro transcriptions. **a** Secondary structure of dGsw illustrating determined pause sites including *t*_1/2_ values using *E*. *coli* polymerase (Supplementary Fig. 6 and Table 4 **b, c**) fractions of ON state from *E. coli* RNAP mediated **b** single-round and **c** multi-round in vitro transcriptions performed in the absence (black) and presence of ligand (gray) at 37 °C, 25 °C, and 4 °C with an NTP concentration of 0.05 mM (**b**) and 0.1 mM (**c**) (Supplementary Fig. 5, 6). Indicated transcription rates are correlated to the time point, at which half of the polymerases have transcribed the corresponding mRNA fragment

For both polymerases, three pause sites (PS1) were determined in time-resolved transcription reactions. The first PS1 is encountered when the aptamer is fully transcribed (Fig. 6a, Supplementary Fig. 7). A decrease in temperature from 37 °C to 4 °C increases the *t*_1/2_ value of PS1 from 3 s to 50 s in the absence of ligand for *E*. *coli* RNAP. The second PS1 is located around nucleotide 116 at the 3′-end of segment T. This second PS1 pauses the transcription complex longer than the first PS1 (24 s at 37 °C). The third PS1 is located at the translation start site. This PS1 is located beyond the regulatory decision point and is, thus, not relevant for the co-transcriptional folding pathway of the riboswitch.

For the *E*.* coli* RNAP, the ligand-dependent ON state fraction is primarily affected by changes in temperature, while variations in the NTP concentration in the range of 0.05–2.3 mM do not affect the transcription rate sufficiently to cause changes in the co-transcriptional folding pathway (Supplementary Fig. 6). In the absence of ligand, the fractions of ON state represent 49–61% of all transcripts, and slightly increases with decreasing temperature (Fig. 6b). For the highest transcription rate (15.1 ± 4.5 nt s^−1^, 37 °C) (Supplementary Fig. 7) the fraction of ligand-independent ON state is 49 ± 12% and for the lowest transcription rate (1.2 ± 0.2 nt s^−1^, 4 °C) the fraction of ON state is 61 ± 9%. In line with riboswitch function, addition of ligand promotes accumulation of nascent transcripts within pathway 3 and decreases the fractions of ON state obtained to 35–43% for transcription reactions performed at 37 °C and 25 °C. For multi-round transcriptions, the effect of ligand addition on the fraction of ON state obtained is significantly more pronounced with 40–50% change in population ratio opposed to ~20% for single-round transcriptions (Fig. 5c). For transcription reactions performed at 4 °C, ligand addition does not cause significant changes in the population of ON state for both single-round and multi-round transcription.

For the *B*. *subtilis* RNAP, transcription rates could only be determined at 37 °C due to insufficient transcription efficiency at lower temperatures. Transcription rates for *B*. *subtilis* RNAP are higher compared to *E*. *coli* RNAP (34.7 ± 30.7 nt s^−1^) (Supplementary Fig. 7). In addition, the decrease in ON state population upon addition of ligand is significantly less pronounced. At an NTP concentration of 2.3 mM, ligand addition only induces a 5% shift in the fraction of ON state. Contrary to the behavior of *E*.* coli* polymerase, a decrease in the NTP concentration to 0.05 mM increases the ligand-induced shift in the fraction of ON state to 21%.

For *E*. *coli* polymerase, the overall regulation efficiency determined by transcription multi-round assays matches remarkably well with values predicted by our simulations of 40–50%, provided transcription reactions were performed close to the physiological temperature of *E*.* coli*. While an NTP-concentration range of 0.05–2.3 mM does not appear to affect transcription rates significantly to influence the co-transcriptional folding pathway of the riboswitch, a reduction in temperature to 4 °C leads to near-complete loss of riboswitch function. Determined transcription rates clearly show a negligibly small decrease in transcription speed from 37 °C to 25 °C, while transcription rates drop below the functional window of the riboswitch (<10 nt s^−1^) as determined by our simulation at 4 °C. In agreement with our simulations, slower transcription rates at 4 °C expand the time span available for PA–T to P^A^T(M) folding and lead to an increase in structures populating the ON state at the regulatory decision point in both absence and presence of ligand. In stark contrast, the *B*. *subtilis* polymerase requires a reduction in NTP concentration to induce transcription termination upon ligand addition. Due to the high percentage of full-length transcripts this effect is likely unrelated to ligand binding or ON state folding, but is linked to the inability of terminator formation due to the rapid elongation by the polymerase. The calculated transcription rates from the in vitro assays are not absolute values for single RNAPs, but instead are composite rates, as they indicate the point in time at which half of the RNAPs completed transcription of an RNA fragment of a specific length. This approximate rate can then be used in the simulations to examine the relationships of the different parameters in the regulation mechanism.

### Discussion

To act as transcriptional regulatory elements, the sequence of riboswitches has to be optimized to fulfill a number of kinetics requirements. Our experimental approach delineates the competition between refolding rates of cis-regulatory RNA and processive transcript elongation. Within the regulatory decisive 2′dG concentration range of 0.1–100 µM for dGsw^26^, ligand association rates for purine aptamer domains of ~10^4^–10^5^ M^−1^ s^−1^ ^27,28^ can effectively direct ligand-binding to either compete with or lag behind bacterial polymerases synthesizing the relevant ligand-binding sequence at rates of 0.5–4.5 s^−1^. Remarkably, PS1 induces transcriptional stalling at the latest possible transcript length with highest ligand binding affinity according to previous results^29^. To determine shifts in population ratios as transcription proceeds further, we present a ligation-based approach that provides access to reversible trapping of metastable states that form within the activation (pathway 1) and repression pathway (pathway 3) of ON function (pathway 2). In the biological context of co-transcriptional folding, single nucleotides are sequentially released to an ensemble of RNA intermediates differing in length that are transcribed at locally varying transcription rates. Real-time monitoring of such folding is experimentally challenging and cannot be accomplished by established methods. In the approach applied here, folding is monitored by immediately releasing a part of the riboswitch expression platform to interact with the aptamer domain. Despite these experimental limitations, we have selected a representative transcription intermediate that most accurately reflects refolding rates of the metastable state on average to obtain high-quality quantitative data. We linked kinetics derived from light-induced release of the stable ON state to unbiased mapping of conformational space^15^ to assess the kinetics of ligand-dependent conformational switch. We further experimentally validated the impact of transcription rates and polymerase dependent transcription properties on the regulation efficiency of the riboswitch using *E*.* coli* and *B*. *subtilis* RNAP.

Our results demonstrate the requirement for a strict synchronization of transcription intervals of metastable states, corresponding transcription rates, and refolding rates, where transcription elongation must not only be matched to refolding and ligand binding kinetics but also to folding delays induced by thermodynamic stabilizations to preserve metastable states within distinct sequence intervals. A significant offset in temperature from physiological conditions leads to quantitative folding to the ON state from both metastable ligand-bound and ligand-free aptamer domains. Sequence-specific variations in transcription rates impact both ON state folding from pathways 1 and 3 equally. Therefore, it is critical that pausing within the expression platform is adapted to the sequence-specific transcription speed of the native polymerase. In a previous study we could already show that the detailed investigation of transcriptional riboswitches requires the native RNA polymerase^30^. Here, we further substantiate this proposal by revealing the difference in co-transcriptional riboswitch folding transcribed by two non-native polymerases. Furthermore, biologically demanding experiments with *M*. *florum* polymerase would be required to compare these results to native 2′dG riboswitch folding. General fluctuations in transcription rates resulting from changes in cellular conditions^31^ may be self-regulated by the negative feedback loop of the riboswitch itself. The kinetic and simulation framework developed here provides an important contribution to the conceptual basis to understand transcriptional riboswitches.

## Methods

### Sample preparation

dGsw^85^ and mutated dGsw^C74U^ constructs with varying length for stopped-flow spectroscopy were prepared by in vitro transcription from PCR products by adjusting the reverse primers accordingly as reported previously^32^. For dGsw^C74U^ constructs, two artificial G’s were incorporated at the 5′-end in the forward primers to enable rapid RNA purification by buffer exchange. dGsw^85^ was transcribed with a 5′-hammerhead ribozyme, exchanged into water, and directly used for ligation reactions.

Photocaged dGsw^86–121^ was prepared via solid phase synthesis in a 17 µmol scale. Ultramild phosphoramidites, chemical phosphorylation reagent I, and (*S*)-NPE-U phosphoramidite (Supplementary Fig. 2) were used and a postsynthetic DEA wash performed. The use of NH_3_:EtOH (3:1) for deprotection primarily generates a byproduct with a mass 53 Da greater than the desired product (Supplementary Table 1). The dGsw^86–121^ fragment could be isolated using 3 mL *t*-BuNH_2_:water (1:3) for 6 hours at 60 °C. The mixture was separated in 10 reaction tubes and the solvent evaporated. Each tube was treated as follows: the 2′-deprotection was performed using 6.5 mL of a mixture of NMP:Et_3_N:Et_3_N·3 HF (3:1.5:2) for 3 hours at 60 °C. After addition of 30 mL *n*-BuOH the mixture was stored at −80 °C for 2 hours and then centrifuged at −8 °C for 1 hour. The supernatant was decanted and the residue purified via anion exchange HPLC (Amersham Bioscience with an UV-900 detector and P-900 pumps) using a Tricorn SOURCE 15Q AEX 10/150 column (gradient: 0–100% 0.35 M LiClO_4_ in water in 17 column volumes, flow: 4 mL min^−1^). The collected oligoribonucleotide was desalted using a Tricorn SOURCE 15 RPC 10/150 column (gradient: 5–40% MeCN in 0.1 M TEAA buffer pH 7 in 9 column volumes, flow: 4 mL min^−1^). The identity of the oligoribonucleotide was confirmed by ESI-MS: mass calculated: 11784.5 Da, found: 11785.7 Da.

Photocaged dGsw^121^ was prepared by enzymatic splinted ligation using T4 RNA ligase 2. Fragments dGsw^85^ (^15^N-G/U) and photocaged dGsw^86–121^ were preannealed to a 31 nt splint (splint:^31^AATAATTAAAGAGTGGTTTTTCACTTATAGT) prior to ligation at 25 °C for 15 min. Ligation reactions were performed with 12.5 µM dGsw^85^, 6.25 µM caged dGsw^86–121^, 10 µM splint^31^, 48 µg mL^−1^ T4 RNA ligase 2 (homemade), 10 mM MgCl_2_, 2 mM ATP, 25 mM DTT in 50 mM Tris-Cl, pH 7.4. dGsw^85^ was applied in 2-fold excess to optimize the turnover for photocaged dGsw^86–121^ (80%). Ligation reactions were incubated at 37 °C for 2 hours followed by overnight incubation at 25 °C, followed by incubation with TURBO DNAse (0.02 U µL^−1^) (Thermo Fisher Scientific) for 8 hours. Ligation reactions were directly applied to HPLC using a Kromasil RP18 10 × 250 column without further concentration and eluted at room temperature (gradient: 0–40% MeCN in 0.1 M TEAA, pH 6.0). Purified photocaged dGsw^121^ was lyophilized and exchanged into NMR buffer (50 mM KCl, 25 mM K_2_HPO_4_/KH_2_PO_4_, pH 6.2). Final yield of photocaged dGsw^86–121^ was 0.6 µmol (54 %).

### NMR-spectroscopy

Time-resolved NMR experiments were performed as reported previously^17,18,33^ in a pseudo-2D experiment using the jump return echo water suppression scheme^34^. Laser irradiation within the NMR tube was triggered via a TLL connection to a laser set up (Paladin Advanced 355-8000). Kinetics of ligand-free samples were recorded after 500 ms and kinetics of ligand-bound RNAs after 1 s of laser irradiation. The signal intensity was maximized by Ernst angle excitation^35^. Kinetics traces of ligand-free samples were averaged over 10 individual experiments and of ligand-bound samples over two individual experiments on 100 µM caged dGsw^121^ samples. ^1^H,^15^N-BEST-TROSY experiments were recorded with modifications according to Brutscher et al^36,37^. NMR experiments were performed on a Bruker 800 MHz spectrometer equipped with a 5-mm *z*-axis TCI-HCN cryogenic probe. For processing of data, Topspin 3.5 (Bruker Biospin) was used.

### Stopped-flow spectroscopy

Stopped-flow measurements were performed on a π*-180 CDF-spectrometer (Applied Photophysics). Folding was monitored by rapid mixing of dGsw^C74U^ (2 µM) with 2-aminopurine 2′-deoxyriboside (2 µM–512 µM) in a 1:1 volume ratio at 25 °C. Both ligand and RNA were provided in NMR buffer with a Mg^2+^ concentration of 3 mM. 2′dAP was excited at 304.5 nm using 2 mm slit widths and fluorescence was monitored at >340 nm. Kinetic traces were averaged over 10–15 single measurements. Fluorescence decays were fitted with single and bi-exponential decays with three and five parameters, respectively (Supplementary Fig. 4). Association (k_on_) and dissociation (k_off_) constants were determined from individual apparent rate constants according to equation (1) (Supplementary Table 3):

1 $$k_{{\rm {app}}} = {k_{\rm {on}}} \times \left[ {2{\prime}\mathrm{dAP}} \right] + {k_{{\rm {off}}}}$$

### Simulations of co-transcriptional folding

Simulations of co-transcriptional folding were performed by kinetic Markov simulations. Simulations were performed as described previously^15^, starting from the aptamer domain fold at transcript length of 113 nt, followed by simultaneous elongation to a transcript length of 137 nt at transcription rates of 10–100 nt s^−1^ and folding to the antiterminator conformations P^A^T(M) and P^A^T(I) with rate constants determined by real-time NMR.

### Time-resolved transcriptions

The DNA template used for *E*. *coli* and *B*. *subtilis* RNAP mediated transcription was composed of the lambdaPR promoter followed by the dGsw-coding sequence (GCAAATCGCGCTGTTAGCGGGCccttgactattttacctctggcggtgataatggttgcATGAATATAAAAGAAACTTATACAGGGTAGCATAATGGGCTACTGACCCCGCCTTCAAACCTATTTGGAGACTATAAGTGAAAAACCACTCTTTAATTATTAAAGTTTCTTTTTATGTCCAAAAGACAAGAAGAAACTTTTTTATTTAGTTGAATTTATAATAAGAGAAAAAGAAAGGATATTATATGGCAAAAATAAAAAACCAATATTACAACGAGTCTGTTTCGCCAATTG). The time-resolved transcriptions were performed as described previously^30^ at 37 °C, 25 °C, and 4 °C with NTP concentrations of 0.05 mM, 0.1 mM, 1 mM, and 2.3 mM. Transcription rates were determined as described previously^38^ and PS1 were analyzed as described by Landick 1996^39^.

### Data availability

The data that support the findings within study are available from the corresponding author upon request.

## Electronic supplementary material

Supplementary Information
