# Supplementary material for: Life times of metastable states guide regulatory signaling in transcriptional riboswitches
Source: Nat Commun. 2018 Mar 5;9:944. doi: 10.1038/s41467-018-03375-w (PMC5838219; doi:10.1038/s41467-018-03375-w)
Supplement: Supplementary file 1 — Supplementary Information [file 41467_2018_3375_MOESM1_ESM.pdf]

Supplementary Information

Life times of metastable states guide regulatory signaling in transcriptional riboswitches

Christina Helmling[1], Dean-Paulos Klötzner[2], Florian Sochor[1], Rachel Anne Mooney[3], Anna Wacker[1], Robert Landick[3], Boris Fürtig[1], Alexander Heckel[2,*], Harald Schwalbe[1,*]

[1]Institute for Organic Chemistry and Chemical Biology, Center for Biomolecular Magnetic Resonance (BMRZ), Johann Wolfgang Goethe-Universität, Max-von-Laue-Straße 9, 60438, Frankfurt, Germany.

[2]Institute for Organic Chemistry and Chemical Biology, Johann Wolfgang Goethe-University Frankfurt, Max-von-Laue-Straße 9, 60438, Frankfurt, Germany.

[3]Department of Biochemistry, University of Wisconsin–Madison, Madison, United States

*Correspondence and requests for materials should be addressed to: schwalbe@nmr.uni-frankfurt.de, heckel@uni-frankfurt.de.

SI1-2: Preparation of photocaged dGsw[121]

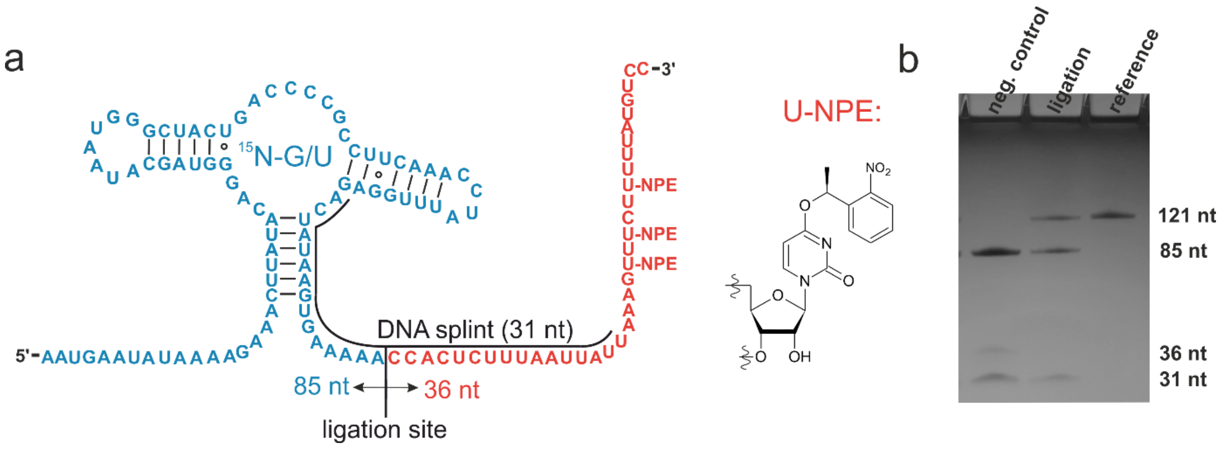

Supplementary Figure 1. Ligation of photocaged dGsw[121].

a) Strategy for preparation of photocaged dGsw[121]. The blue fragment was prepared by *in vitro* transcription in ^{15}N G/U isotope labeled form and the red fragment by solid phase synthesis implementing three NPE (1-nitro-phenylethyl) protection groups. The two fragments were annealed to a 31 nt DNA splint and ligated by enzymatic splinted ligation with T4 RNA ligase 2. b) 12% polyacrylamide gel of the ligation showing a lane for the negative control, the ligation reaction, and isolated dGsw[121] as reference. Individual fragments for ligation splint[31], dGsw[86-121], dGsw[85] and the reference construct dGsw[121] are indicated accordingly.

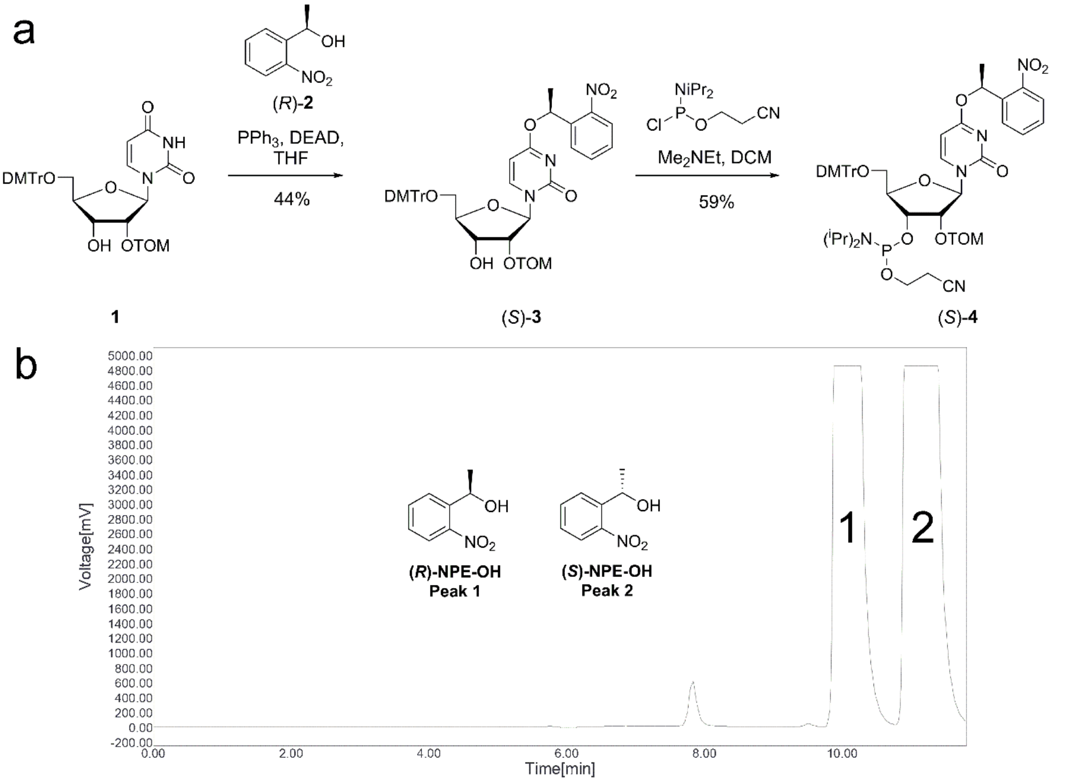

Supplementary Figure 2. Synthesis of (*S*)-NPE-U phosphoramidite (*S*)-4.
a) (*S*)-NPE-U phosphoramidite (*S*)-**4** was prepared according to C. Höbartner et al.[1] except the isolation of the pure enantiomer (*R*)-**2**. b) Elution profile of the racemic mixture of (*R*,*S*)-1-(2-Nitrophenyl)ethanol. Enantiomers were detected at 254 nm. The racemic mixture of (*R*,*S*)-**2** was prepared as described in the literature.[2] The pure enantiomer (*R*)-**2** was isolated via preparative chiral HPLC using a CHIRALPAK® IA column (particle size: 5 µm, dimensions: 20 mm x 250 mm) on an HPLC from Young Lin Instruments with SP930D pumps and a UV730D detector. An isocratic flow (10 mL/min) of a mixture of *n*-Hexan/EtOH (8:2) was used. 90 mg of the racemic mixture were injected per run. The absolute configuration of the two species was determined by measuring the specific rotation.

Supplementary Table 1. Tested analytical deprotection conditions during the purification of the photocaged dGsw$^{86\text{-}121}$ fragment.

Entry	*Treatment with DEA (10% in MeCN)[a]*	*Deprotection with NH_3:EtOH (3:1)*	*2′ Deprotection with NMP:Et_3N:Et_3N·3 HF (3:1.5:2)*	*Detected Mass*
1	/	2 h, rt	90 min, 60 °C	M, M+53
2	/	4 h, rt	90 min, 60 °C	M, M+53
3	/	21 h, rt	90 min, 60 °C	/
4	1 min	2 h, rt	90 min, 60 °C	M, M+53
5	5 min	2 h, rt	90 min, 60 °C	M, M+53
6	15 min	2 h, rt	90 min, 60 °C	M, M+53
7	/	4 h, 55 °C	90 min, 60 °C	M
	Treatment with DEA (10% in MeCN)[a]	*Deprotection with t-BuNH_2:water (1:3)*	*2′ Deprotection with NMP:Et_3N:Et_3N·3 HF (3:1.5:2)*	*Detected Mass*
8	/	6 h, 60 °C	90 min, 60 °C	M

[a] An analytical amount of fully protected oligoribonucleotide attached to CPG was placed in a blank synthesizer column with Luer fittings and treated with a flow (approx. 1 mL/min) of DEA (10% in MeCN).

Analytical deprotection with NH_3:EtOH (3:1) led primarily to the formation of an undesired byproduct with a mass greater by 53 Da compared to the expected product mass. The byproduct could not be separated from the product via HPLC. The use of *t*-BuNH_2:water (1:3) at 60 °C for 6 hours prevented byproduct formation and led the majority of the photolabile protecting groups intact.

SI3: Kinetic traces and rates derived from individual imino protons

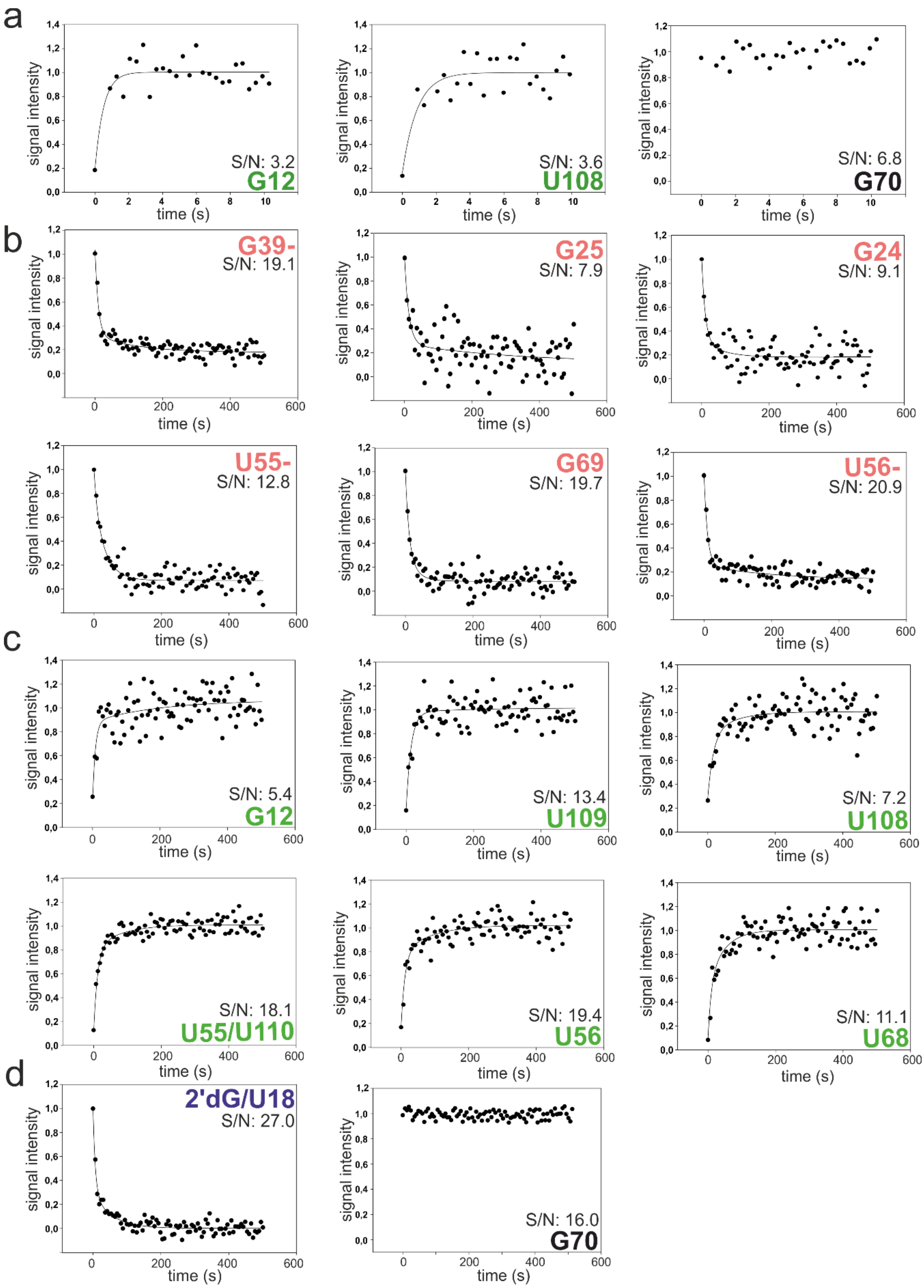

Supplementary Figure 3. Individual kinetic traces obtained from real-time NMR experiments. a) isolated reporter signals G12 and U108 for PT helix formation in the absence of ligand and for the reference signal G70, b) isolated reporter signals for P2 and P3 dissociation in the presence of ligand and c) isolated reporter signals for P2/PT helix formation in the presence of ligand, d) dissociation of 2'dG and U18 in the presence of ligand and reference signal G70. Transients were fit with single ($y(t)=y_0+a(1-e^{-bt})$) and double exponential functions $y(t)=y_0+a(1-e^{-bt})+c(1-e^{-dt})$ or $y(t)=y_0+a*e^{-bt}+c*e^{-dt}$.

Supplementary Table 2. F-values derived from an F-test comparing single-exponential and double-exponential fits. Critical F-values to be considered are F(0.01,2,62)=5.0 and F(0.05,2,62)=3.2 (-2’dG), and F(0.01,2,82)=4.8 and F(0.05,2,82)=3.1 (+2’dG). For G12 and U108, the data could not be reliably fit with a double-exponential fit. In the presence of ligand, only individual kinetic traces with poor S/N are below critical F-values.

**-2’dG**	**G12**	**U108**	**P0**	**G25I**				
**F**	-	-	2.44	0.13				
**+2’dG (rise)**	**G12**	**U109**	**U108**	**U55/U110**	**U56**	**U68**	**P0/P3**	
**F**	3.85	1.96	2.58	5.69	5.08	2.18	17.51	
**+2’dG (decay)**	**G39**	**G25**	**G24**	**U55**	**U68**	**U56**	**P2/P3**	**2’dG/U18**
**F**	11.15	1.47	0.76	5.27	5.45	7.11	19.35	10.57

SI4: Ligand binding kinetics derived from stopped-flow spectroscopy

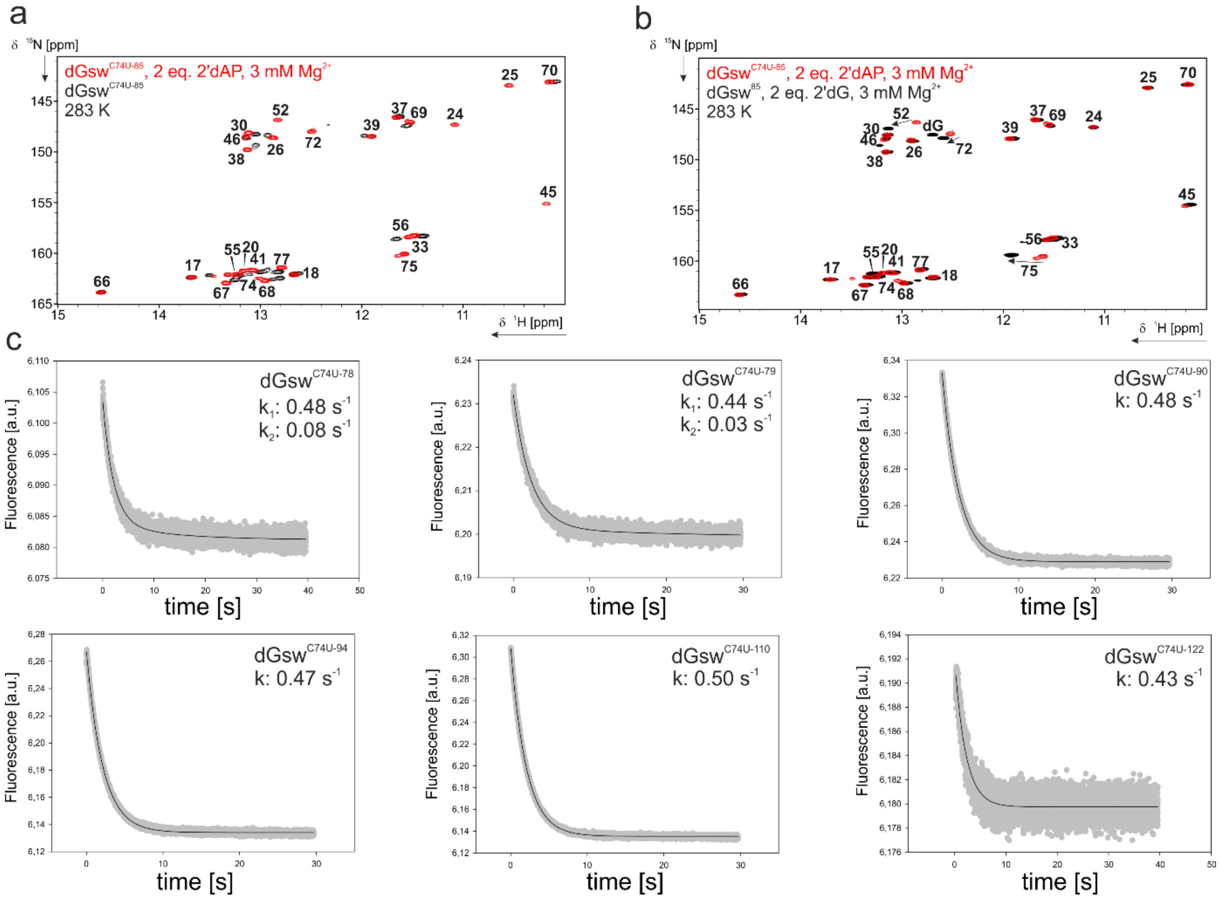

Supplementary Figure 4. Results of stopped-flow fluorescence spectroscopy. a) Overlay of $^{1}H,^{15}N$-TROSY spectra of dGsw$^{C74U-85}$ recorded in the absence (black) and presence of 2 eq. of 2'dAP (red) and 3 mM Mg^{2+} at 800 MHz and 283 K. b) Overlay of $^{1}H,^{15}N$-TROSY spectra of dGsw$^{C74U-85}$ (red) and dGsw^{85} (black) recorded at 800 MHz and 283 K. Arrows indicate chemical shift perturbations caused by the mutation in the binding pocket. c) Kinetic traces of 1 µM of dGsw$^{C74U-78}$, dGsw$^{C74U-79}$, dGsw$^{C74U-90}$, dGsw$^{C74U-94}$, dGsw$^{C74U-110}$ and dGsw$^{C74U-122}$ mixed with 24 eq. of 2'dAP.

The overlay of $^{1}H,^{15}N$-TROSY spectra in Figure S4a of dGsw^{C74U} recorded in the absence and presence of 2'dAP at 3 mM Mg^{2+} shows that the 2'dAP binds to the mutated aptamer domain and adopts a homogeneous ligand-bound state. Ligand binding can be monitored by the appearance of reporter signals G24, G25, G52, and U66. The overlay of $^{1}H,^{15}N$-TROSY spectra in Figure S4b directly compares 2'dAP-bound dGsw^{C74U} to 2'dG-bound dGsw. Mutations in the binding pocket cause chemical shift perturbations of G52, G72, and U75 near the mutation site, but the majority of signals do not shift and verify structural homology.

Figure S4c shows kinetic traces recorded at 24 eq of 2'dAP for different transcript lengths. Fluorescence quenching is significantly decreased for dGsw$^{C74U-78}$, dGsw$^{C74U-79}$ and dGsw$^{C74U-122}$. For dGsw$^{C74U-122,}$ the low degree in quenching can be correlated to its low binding affinity determined by NMR-spectroscopy. dGsw$^{C74U-78}$, dGsw$^{C74U-79}$ contain a truncated P1 helix and the decrease in fluorescence quenching may be a result of incomplete stacking in P1. The bi-exponential behavior of dGsw$^{C74U-78}$ and dGsw$^{C74U-79}$ may describe fast association of the ligand followed formation of a compact ligand binding pocket including tertiary loop-loop interactions, which may occur on a slower timescale in truncated P1 constructs. Apparent rate constants obtained at different ligand concentrations including k_{on}, k_{off}, and K_D values are listed in Table S3.

Supplementary Table 3. Apparent rate constants k_{app} for selected dGsw^{C74U} constructs (1µM) at six different ligand concentrations determined by single and bi-exponential decay and derived ligand association rates k_{on}, ligand dissociation rates k_{off} and equilibrium dissociation constant K_D.

	k_{app} [s^{-1}]						k_{on} [$M^{-1}s^{-1}$]	k_{off} [s^{-1}]	K_D [µM]
[2'dAP] →	16 µM	24 µM	32 µM	48 µM	64 µM	128 µM			
dGsw$^{C74U-78}$	0.436<br>0.016	0.477<br>0.080	0.492<br>0.028	0.476<br>0.047	0.502<br>0.211	0.54<br>-	$1.0*10^3$	0.43	430.0
dGsw$^{C74U-79}$	0.412<br>0.026	0.436<br>0.029	0.442<br>0.042	0.486<br>0.085	0.483<br>0.045	0.552<br>0.207	$1.3*10^3$	0.40	307.7
dGsw$^{C74U-80}$	0.429	0.466	0.503	0.583	0.671	0.967	$5.0*10^3$	0.35	70.0
dGsw$^{C74U-85}$	0.373	0.393	0.434	0.518	0.685	0.948	$4.7*10^3$	0.36	76.6
dGsw$^{C74U-90}$	0.445	0.481	0.525	0.603	0.688	0.922	$8.9*10^3$	0.37	35.6
dGsw$^{C74U-94}$	0.423	0.474	0.520	0.617	0.726	1.107	$6.0*10^3$	0.34	56.6
dGsw$^{C74U-96}$	0.446	0.466	0.520	0.602	0.696	0.968	$5.3*10^3$	0.35	66.0
dGsw$^{C74U-100}$	0.441	0.485	0.527	0.614	0.707	0.945	$5.4*10^3$	0.36	66.7
dGsw$^{C74U-110}$	0.448	0.498	0.538	0.621	0.705	0.995	$5.4*10^3$	0.36	66.7
dGsw$^{C74U-122}$	0.480	0.428	0.420	0.477	0.571	0.936	$5.5*10^3$	0.23	41.8

SI5: Markov simulations of co-transcriptional folding

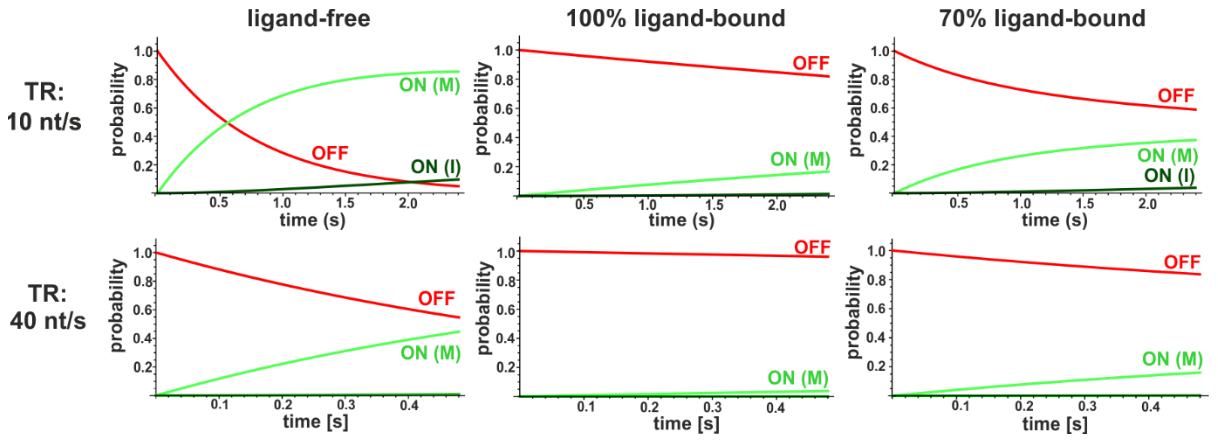

Supplementary Figure 5. Simulations of co-transcriptional folding. Exemplary simulations performed at a transcription rates of 10 nt/s and 40 nt/s in case the ligand is not bound to the aptamer domain before nucleotide 113 is synthesized (ligand-free); ligand binding is completed by 70% (70% ligand-bound) or 100% (100% ligand-bound) by the time nucleotide 113 is synthesized.

SI6-7: Multi-round and single-round transcription reactions with *E.coli* and *B.subilits* RNAP

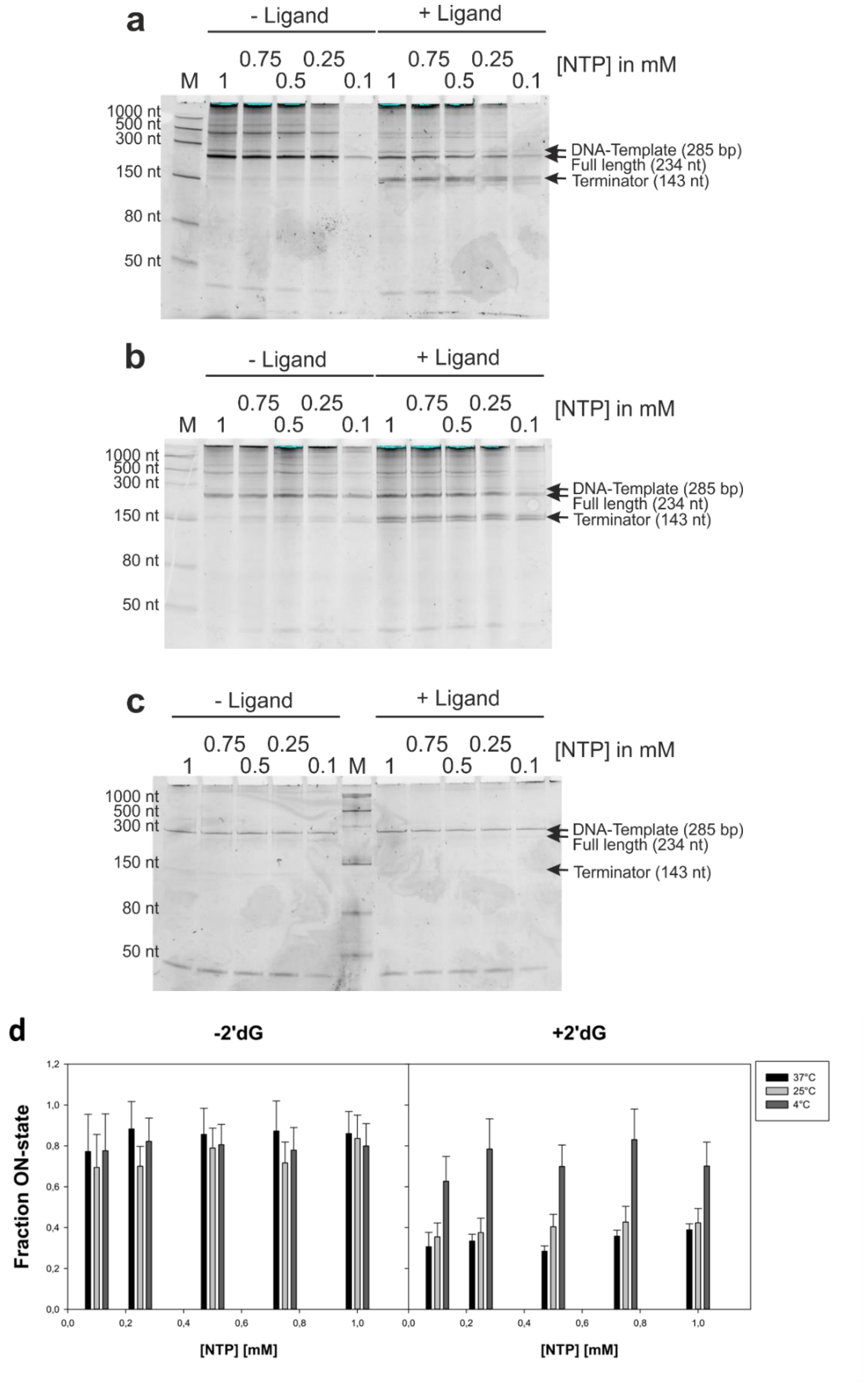

Supplementary Figure 6. *E. coli* RNAP mediated transcritpions. a-c) Multi-round transcription reactions performed at 37 °C (a), 25 °C (b) and 4 °C (c) in the absence (- Ligand) and presence (+ Ligand) of ligand. The NTP-concentration was varied from 0.1-1 mM. The DNA-template, the full-length transcript and the terminator transcript are indicated with the respective lengths of the constructs. The used marker is the low range ssRNA marker from NEB (Frankfurt, Germany). d) Fractions of ON-state derived from multi-round transcriptions shown in a-c.

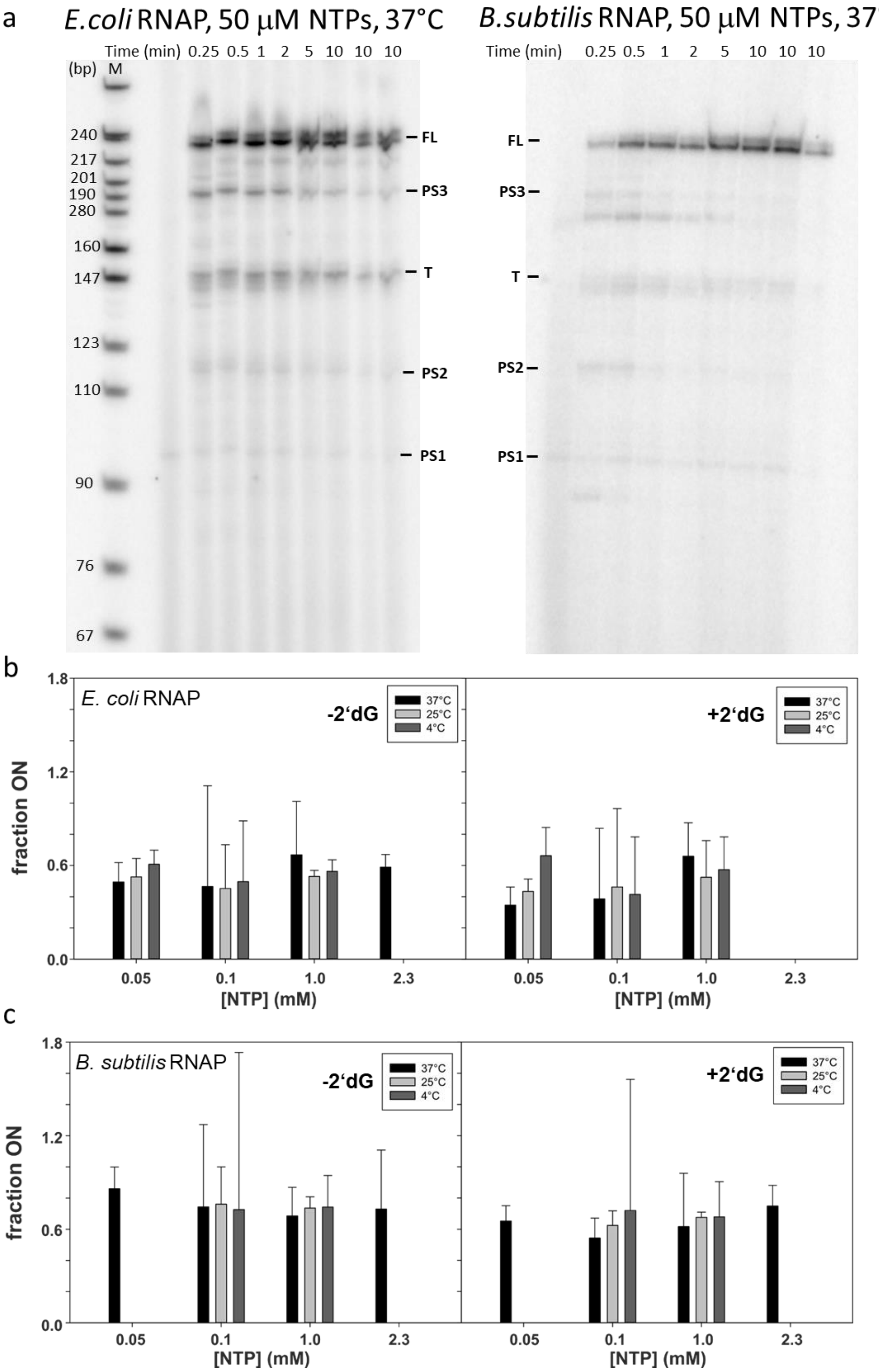

Supplementary Figure 7. Time resolved transcription reactions with *E. coli* RNAP *and B.subtilis RNAP.* a) Single-round transcription reactions performed at 37 °C at an NTP concentration of 50 µM. b,c) Fraction ON-state derived from the single round time resolved transcriptions for the *E. coli* (b) and the *B. subtilis* RNAPS (c) at time point 10 min. The fraction of ON-state was calculated at NTP-concentrations varying from 0.05-2.3 mM and at 4 °C, 25 °C and 37 °C. For the *E. coli* RNAP, the fraction ON-state at 37 °C and 2.3 mM NTP-concentration could only be determined in the absence of ligand. For the *B. subtilis* RNAP, the fraction ON-state at extreme NTP-concentrations could only be determined at 37 °C

Supplementary Table 4. Transcription rates and pause site $t_{1/2}$ values derived from gels shown in figure S7 at an NTP concentration of 50 μM calculated as described previously.[3]

**Transcription rates**				
RNA	*E. coli*, 37 °C	*B. subtilis*, 37 °C	*E. coli*, 25 °C	*E.coli*, 4 °C
FL	9.6±2.2	17.9±5.9	6.8±1.7	0.8±0.03
T	15.1±4.5	34.7±30.7	12.2±4.1	1.2±0.2

**Pause site $t_{1/2}$ (s)**				
RNA	*E. coli*, 37 °C	*B. subtilis*, 37 °C	*E. coli*, 25 °C	*E.coli*, 4 °C
190-202 (PS3)	177.1	250.9	62.7	1505.1
116 (PS2)	23.5	39.6	11.3	143.3
91-101 (PS1)	3.1	17.7	7.2	50.2

References

1. Höbartner, C. & Silverman, S. K. Modulation of RNA Tertiary Folding by Incorporation of Caged Nucleotides. *Angew. Chemie Int. Ed.* **44,** 7305–7309 (2005).

2. Corrie, J. E. T., Reid, G. P., Trentham, D. R., Hursthouse, M. B. & Mazid, M. A. Synthesis and absolute stereochemistry of the two diastereoisomers of P ^{3} -1-(2-nitrophenyl)ethyl adenosine triphosphate ('caged' ATP). *J. Chem. Soc., Perkin Trans. 1* 1015–1019 (1992). doi:10.1039/P19920001015

3. Landick, R., Wang, D. & Chan, C. L. Quantitative analysis of transcriptional pausing by Escherichia coli RNA polymerase: his leader pause site as paradigm. *Methods Enzymol.* **274,** 334–353 (1996).
